# Systemic adverse events after screening of retinopathy of prematurity with mydriatic

**DOI:** 10.1371/journal.pone.0256878

**Published:** 2021-09-09

**Authors:** Shumpei Obata, Taku Imamura, Masashi Kakinoki, Takahide Yanagi, Yoshihiro Maruo, Masahito Ohji

**Affiliations:** 1 Department of Ophthalmology, Shiga University of Medical Science, Otsu, Japan; 2 Department of Pediatrics, Shiga University of Medical Science, Otsu, Japan; Metrohealth Medical Center, UNITED STATES

## Abstract

**Purpose:**

To evaluate systemic adverse events after screening for retinopathy of prematurity (ROP) performed with mydriatic.

**Methods:**

This was a retrospective case series study. Medical records of consecutive patients who underwent screening for ROP with 0.5% phenylephrine and 0.5% tropicamide eyedrops were retrospectively reviewed. The score of abdominal distention (0–5), volume of milk sucked and volume of stool, along with systemic details (pulse and respiration rates, blood pressure and number of periods of apnea) were collected at 1 week and 1 day before ROP examination, and at 1 day after examination. Results were compared between the days before and after examination. Correlation between body weight at the time of examination and the score of abdominal distention was examined. The numbers of infants with abdominal and/or systemic adverse events were compared between pre- and post-examination periods.

**Results:**

Eighty-six infants met the inclusion criteria. The score of abdominal distention increased from 2.0 at 1 day before examination to 2.3 at 1 day after examination (p = 0.005), and the number of infants who had worsened abdominal distension increased after examination (p = 0.01). Infants with lower body weight had a higher score of abdominal distention (p < 0.0001, r = −0.57). The number of infants with reduced milk consumption increased after examination (p = 0.0001), as did the number of infants with decreased pulse rate (p = 0.0008).

**Conclusions:**

Screening for ROP with mydriatic may have adverse effects on systemic conditions. Infants should be carefully monitored after ROP screening with mydriatic.

## Introduction

Retinopathy of prematurity (ROP) is a leading cause of childhood blindness world-wide [1]. Screening preterm infants at risk of ROP requires serial funduscopic examinations using mydriatic eyedrops; 0.5–1.0% tropicamide and/or 0.5–10% phenylephrine and/or 0.2–1.0% cyclopentolate eyedrops. Some complications from mydriatic eyedrops have been reported in preterm infants. Systemic absorption of mydriatic eyedrops has been associated with cardiovascular, respiration and gastrointestinal adverse effects [2,3]. ROP screening using cyclopentolate may be associated with increased gastric residuals and delayed gastric emptying [4,5]. In Japan, 0.5% phenylephrine and 0.5% tropicamide eyedrops are commonly used for ROP screening. There have been reports about systemic adverse events from other mydriatics or from different doses of combined phenylephrine and tropicamide eyedrops other than case reports.[4–6]. However, to the best of our knowledge, there is one report regarding abdominal adverse effects after ROP screening with 0.5% phenylephrine and 0.5% tropicamide eyedrops. This previous report evaluated necrotizing enterocolitis and gastric residual as abdominal adverse effects [7]. In this report, we evaluated score of abdominal distention, volume of milk sucked, volume of stool as abdominal distention, and pulse rate, respiration rate, blood pressure and number of episodes of apnea as systemic adverse events from ROP screening with 0.5% phenylephrine and 0.5% tropicamide eyedrops between before and after ROP screening.

## Methods

This study protocol was approved by the Institutional Review Board (IRB)/Ethics Committee Shiga University of Medical Science (Otsu, Japan). For this type of retrospective study, an opt-out consent process was used at our institution. All data were fully anonymized before we accessed them. Patients’ medical records were accessed from July 2019 to December 2020. This study adhered to the tenets of the Declaration of Helsinki. Medical records of consecutive preterm infants who were screened for ROP between June 2016 and March 2018 at the Shiga University of Medical Science Hospital Neonatal Intensive Care Unit (NICU) were retrospectively reviewed. The ROP screening was performed following the guidelines proposed by the American Academy of Ophthalmology, and the Association for Pediatric Ophthalmology and Strabismus [8], with some modifications. ROP screening was performed after instilling 0.5% phenylephrine and 0.5% tropicamide eyedrops 3 times at a 15-minute interval. For infants with abdominal trouble before ROP screening, we used 0.25% phenylephrine and 0.25% tropicamide eyedrops by diluting the eyedrops. Infants whose ROP screening was performed with the lower-dose eyedrops were excluded from the present study. Gestational age, birth weight, gender and time of first ophthalmic examination were collected from medical charts. Score of abdominal distention (0–5), volume of milk sucked, volume of stool, and pulse rate, respiration rate, blood pressure and number of episodes of apnea (cessation of respiration for ≥20 s, or cessation of respiration of any duration accompanied by bradycardia (heart rate <100/min) and/or cyanosis was defined as an episode) were collected from the medical records of patients at 1 week and 1 day before ROP examination, and 1 day after examination. We defined the period from 1 week to 1 day before as the “period before examination” and the period from 1 day before to 1 day after as the “period after examination.” We also defined 1 day before examination as the “baseline”. The abdominal-distention score was defined as follows, and was evaluated by nurses in the NICU: 0 = no abdominal distension; 1 = slight abdominal distension; 2 = abdominal distension that was mild but soft; 3 = abdominal distension that was moderate but soft; 4 = abdominal distension that was moderate and slightly hard; and 5 = severe abdominal distension that was hard. Mean results were compared for 1 week before, 1 day before and 1 day after examination. However, analysis of only the mean data might mask the effects on individual infants. Therefore, we also analyzed the means of individual measurements and the numbers of infants who changed between before versus after examination.

Correlation between the score of abdominal distention and body weight at screening was performed.

Statistical analyses were performed using GraphPad Prism 6 software (GraphPad Software, Inc., La Jolla, CA, USA). The results were expressed as the mean ± standard deviation (SD) for continuous variables and as proportions (%) for categorical variables. The Wilcoxon test was used for comparisons of changes between 1 week before, baseline and 1 day after examination. Spearman correlation coefficients were assessed to determine the relationships between body weight at ROP screening and abdominal-distention score. McNemar’s test was used to compare the number of infants who had a change in abdominal and systemic adverse effects between the period before examination and the period after examination. P-values < 0.05 were considered significant.

## Results

Eighty-nine babies were screened for ROP during the period under investigation. All data are provided in S1 File. Pupil dilation was performed with 0.5% phenylephrine and 0.5% tropicamide eyedrops in 86 patients (96.6%). The eyedrops were diluted by half in the other 3 patients, who had abdominal trouble before ROP screening (3.4%). Eighty-six infants who underwent screening of ROP in our hospital met the inclusion/exclusion criteria and were included in this study. Forty-two patients were female (49%). Gestational ages ranged from 24–35.1 weeks post-menstrual age (PMA) (mean: 32 weeks), and weights ranged from 654–2710 g (mean ± SD: 1606 ± 438.7 g) at birth. PMAs at time of ROP screening ranged from 29.7–39.3 weeks (mean: 35.3 weeks), and body weight ranged from 856–2674 g (mean ± SD: 1871 ± 442.4 g).

### Abdominal distention

**Fig 1:**
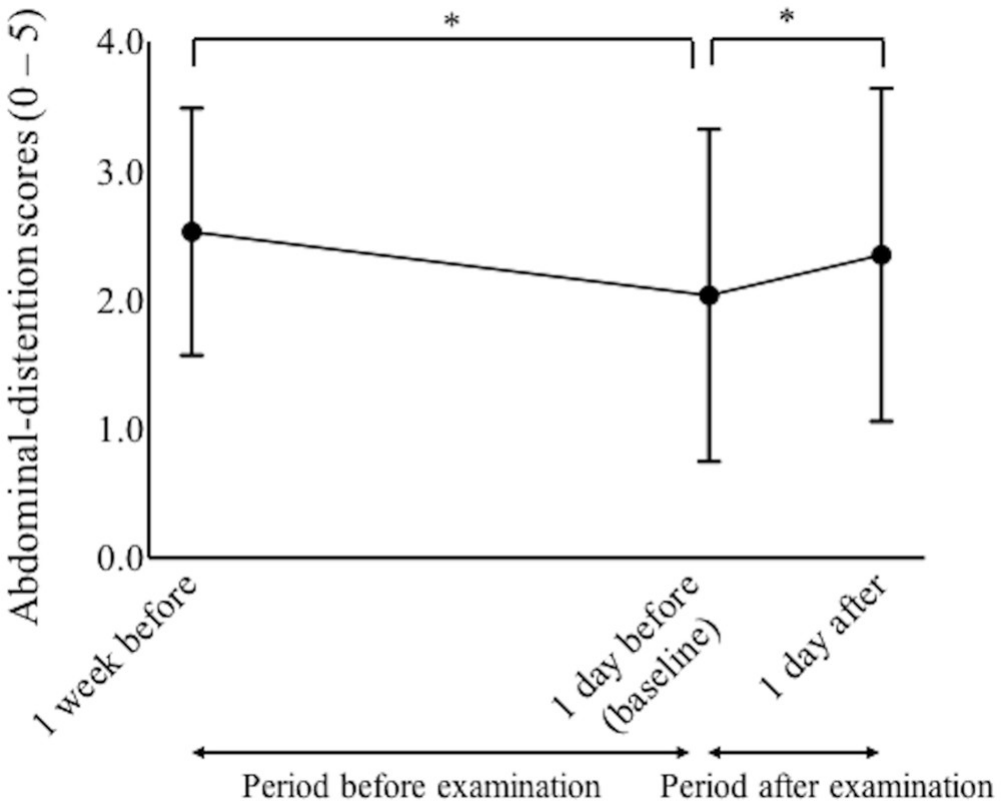
The scores of abdominal distention from 1 week before examination to 1 day after screening for retinopathy of prematurity. Low scores of abdominal distention represent less abdominal distention. *Statistical difference (p < 0.05, paired-t tests with Bonferroni correction).

The abdominal-distention scores decreased significantly from 1 week to 1 day before ROP examination. The abdominal-distention scores then increased significantly from 2.0 at baseline to 2.3 at 1 day after examination (p = 0.005 for both comparisons, paired-t tests with Bonferroni correction, see Fig 1).

Compared with their scores at 1 week before examination, the scores had worsened in 7 patients (8.1%) at 1 day before examination. However, compared with their baseline score, the score worsened in 21 patients (24.4%) at 1 day after examination. Compared with the period before examination (from 1 week before examination to baseline), there was a significant increase in the number of infants whose abdominal score worsened during the period after examination (from baseline to 1 day after examination) (p = 0.01, McNemar’s test).

**Fig 2:**
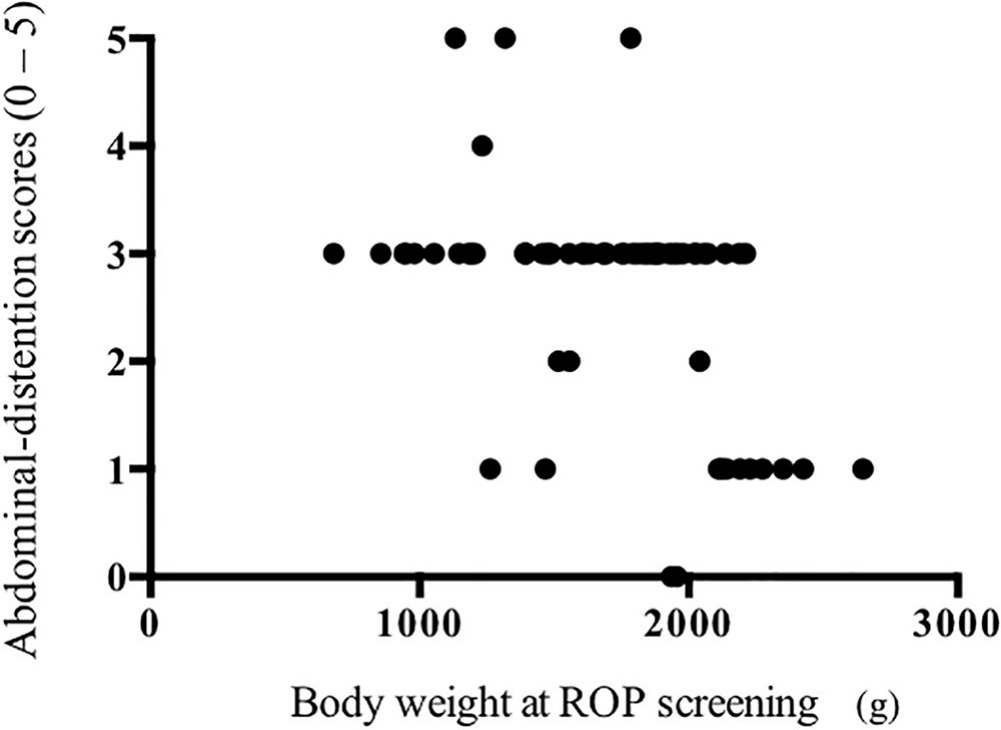
Correlation between body weights at retinopathy of prematurity (ROP) examinations and the scores of abdominal distention.

Infants with lower body weight at the time of their ROP screening had higher scores of abdominal distention at that time (p < 0.0001, r = −0.57 Spearman rank correlation coefficient, see Fig 2).

### Other abdominal adverse events

**Fig 3:**
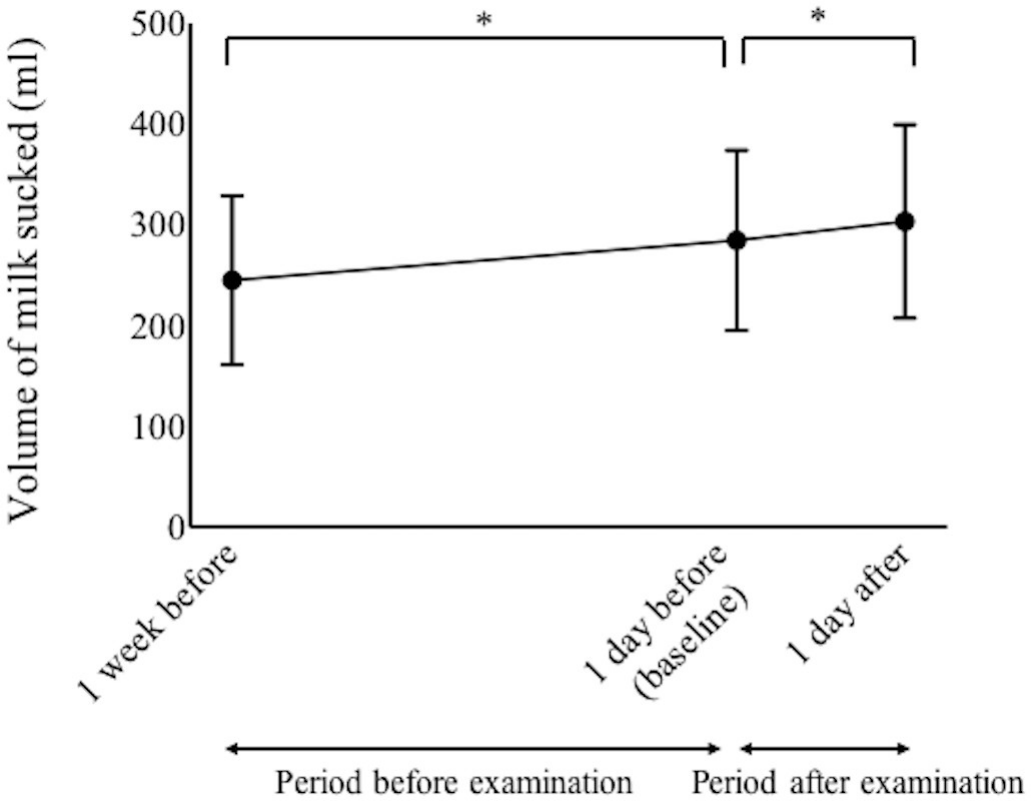
The volume of milk sucked, per infant per day, from 1 week before examination to 1 day after examination. *Statistical difference (p < 0.05, paired-t tests with Bonferroni correction).

Details regarding the volumes of milk consumed were collected for the 86 neonates. The mean amount of milk sucked per infant per day increased significantly from 284.5 ± 89.4 ml at 1 day before examination to 303.4 ± 96.1 ml at 1 day after examination (p < 0.0001, paired-t test, Fig 3).

However, compared with the amount of milk sucked at 1 week before examination, the amount decreased in 6 patients (6.9%) at 1 day before examination. Compared with the amount of milk sucked at 1 day before examination, the volume decreased in 20 patients (23%) at 1 day after examination. Compared with the period before examination (from 1 week before examination to baseline), there was a significant increase in the number of infants who decreased the amount of milk that they sucked during the period after examination (from baseline to 1 day after examination) (p = 0.0001, McNemar’s test).

**Fig 4:**
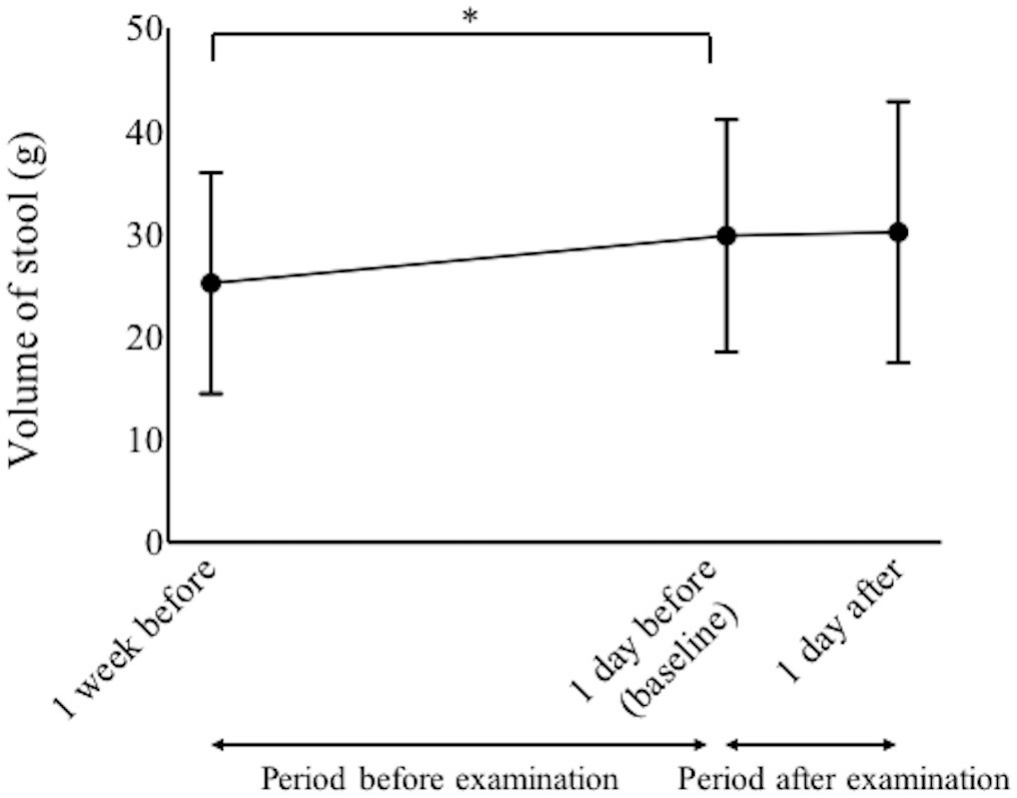
The daily stool volumes measured from 1 week before examination to 1 day after examination. *Statistical difference (p < 0.05, paired-t tests with Bonferroni correction).

The measured volumes of stool were collected from the records of 66 of the neonatal infants. The volume of stool increased significantly from 24.5 ± 11.0 g at 1 week before examination to 29.8 ± 11.3 g at 1 day before screening (p = 0.0082, paired-t test, Fig 4), but the volume of stool did not significantly increase from 29.8 ± 11.3 g at 1 day before screening to 30.1 ± 12.7 g at 1 day after examination (p > 0.05, paired-t test, Fig 4).

Compared with the period before examination (from 1 week before examination to baseline), there was no significant change in the number of infants who decreased the volume of stool during the period after examination (from baseline to 1 day after examination) (p > 0.05, McNemar’s test). None of the neonatal infants developed ileus during the examination period.

### Systemic vital data

**Fig 5:**
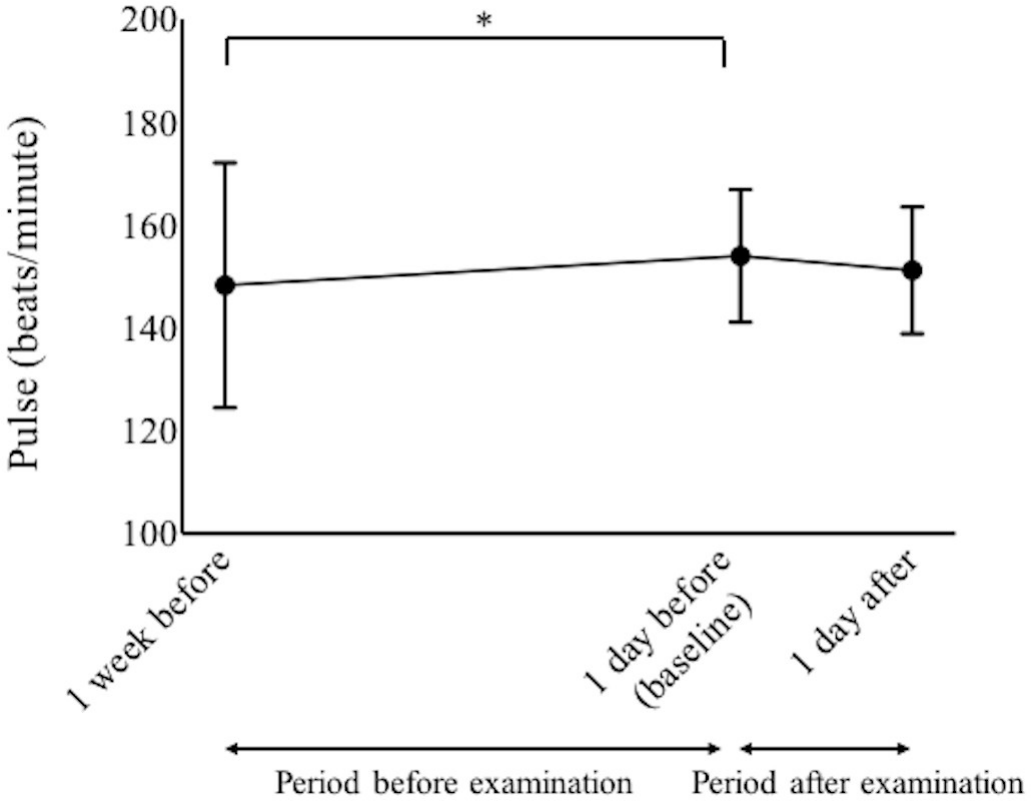
Pulse rates from 1 week before examination to 1 day after examination. *Statistical difference (p < 0.05, paired-t tests with Bonferroni correction).

Pulse rates increased significantly from 148 ± 23.8 beats per minute (bpm) at 1 week before examination to 154 ± 12.9 bpm at 1 day before examination (p = 0.02, paired-t test). However, pulse rates did not increase from baseline to 1 day after examination (Fig 5).

Compared with the pulse rate at 1 week before examination, the rate had decreased in 33 patients (38.8%) and increased in 52 patients at 1 day before examination. Compared with rates at 1 day before examination, the rate decreased in 47 patients (56.4%) and increased in 38 patients at 1 day after examination. Compared with the period before examination (from 1 week before examination to baseline), there was a significant increase in the number of infants whose pulse rate decreased during the period after examination (from baseline to 1 day after examination) (p = 0.0008, McNemar’s test).

**Fig 6:**
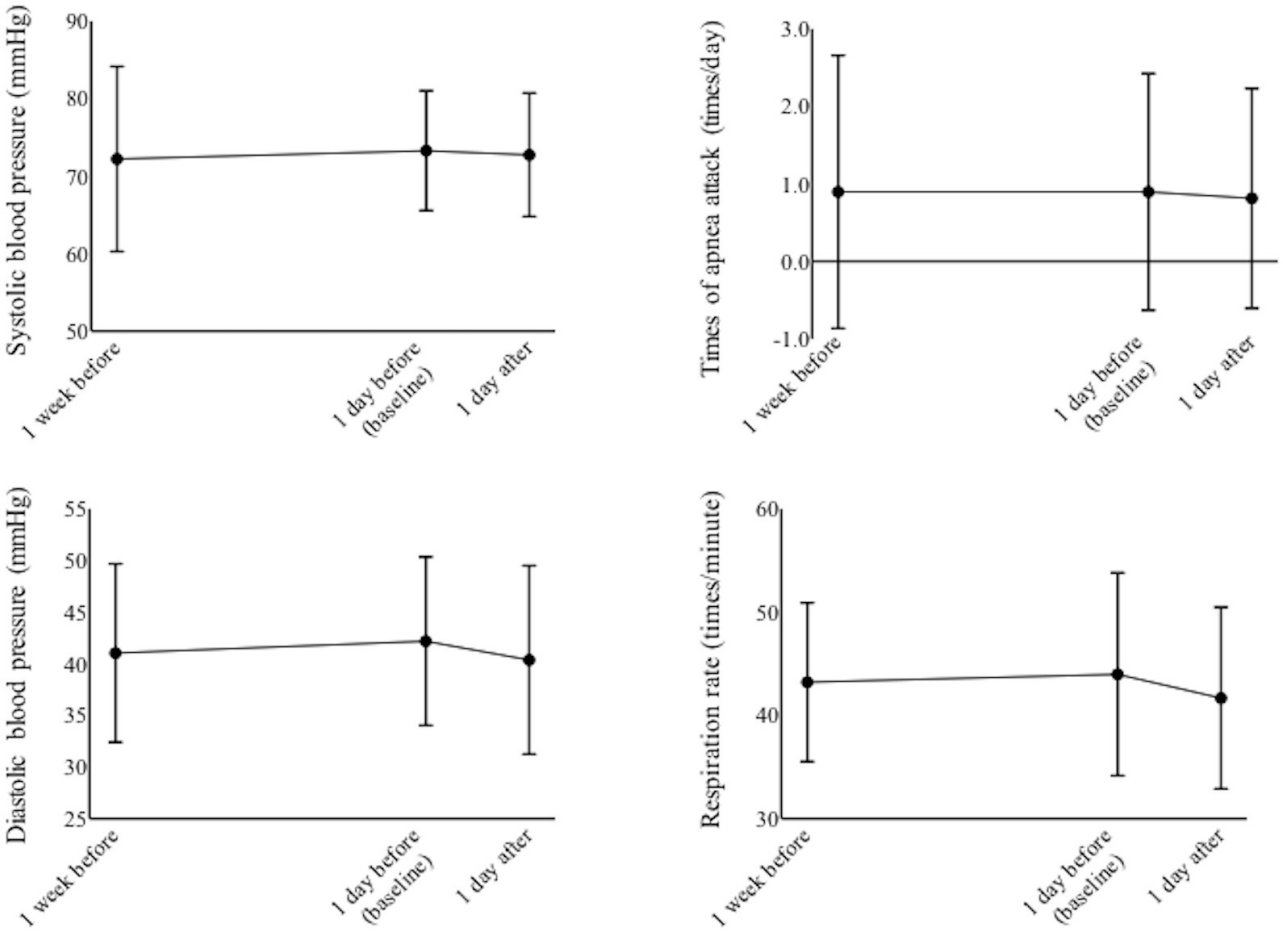
Systolic and diastolic blood pressure, number of periods of apnea and respiration rates from 1 week before examination to 1 day after examination.

Respiration rate, blood pressure (systolic and diastolic) and episodes of apnea did not change significantly through the study period (p > 0.07, paired-t tests, Fig 6).

## Discussion

In the current study, the abdominal-distention scores significantly increased and the number of neonatal infants who consumed a reduced amount of milk increased after ROP screening with 0.5% phenylephrine and 0.5% tropicamide eyedrops. To the best of our knowledge, our study is the first to evaluate abdominal adverse events (abdominal-distension scores, volume of milk sucked and the volume of stool) after ROP screening with this combination of eyedrops between before and after ROP screening. In Japan, 0.5% phenylephrine and 0.5% tropicamide eyedrops are commonly used for ROP screening. Jiang et al. reported that ROP screening with 0.5% phenylephrine and 0.5% tropicamide was associated with residual gastric contents and necrotizing enterocolitis using a different evaluation item from the current study [7]. However, there are some reports in which other mydriatics, or different doses of phenylephrine-and-tropicamide, were used.

In the previous reports with different doses of phenylephrine-and-tropicamide, abdominal adverse effects have been reported. Degirmencioglu et al. reported a case of an infant with very low birth weight who developed significant abdominal symptoms (mimicking ileus), with discontinuation of oral feeding, after ocular instillation of 2.5% phenylephrine and 0.5% tropicamide eyedrops for routine examination of ROP [9]. In this previous report, the concentration of phenylephrine was higher than that in the current study. Even relatively thin concentrations of 0.5% phenylephrine and 0.5% tropicamide eyedrops may have adverse effects on abdominal adverse effects.

In the previous reports with other mydriatics, abdominal adverse effects have been reported. A 2-month-old girl had episodes of apnea, vomiting and distension after a screening for ROP with cyclopentolate 0.5% and phenylephrine 2.5% [10]. Bonthala et al. reported that ocular instillation of 0.2% cyclopentolate and 1.0% phenylephrine eyedrops inhibited duodenal motility and delayed gastric emptying in 11 infants; this might have been caused by a reduction in peristaltic movement of the gastrointestinal tract [5]. ROP screening using cyclopentolate, therefore, might be associated with increased gastric residuals and cyclopentolate was detected in the blood of infants, whereas phenylephrine was not detected in the blood [4]. In another report, the incidence of gastric residual increased after ROP screening with another type and/or dose of mydriatic [11]. In these reports, cyclopentolate was used for ROP screening; therefore, it may cause abdominal adverse effects.

In the present study, neonatal infants with lower body weight at the ROP screening had higher scores of abdominal distention. However, because those infants also had higher scores of abdominal distention *before* ROP screening, we should be careful about attributing the abdominal adverse effects to the mydriatic (alone). Jiang et al. reported that the incidence of gastric residuals increased after ROP screening in infants with postconceptional age < 31 weeks [7]. Gronlund et al. reported that, in neonatal lambs, the sympathetic nervous system is a major regulator of cardiovascular interactions and that the autonomic nerves are immature [11]. The immaturity of autonomic nerves in neonatal infants might contribute to abdominal adverse effects.

In the current study, compared with the period before examination, there was a significant increase in the number of infants with decreased pulse rate during the period after examination. In some previous reports, pulse rate did not significantly change after ROP screening [5,12–15]. However, Khoo et al. reported that pulse rates decelerated below the baseline values after ROP screening [16]. Rosales et al. stated that stress and pain during ophthalmic examinations are known to precipitate apnea and bradycardia [17]. Stress and pain during ophthalmic examination may affect pulse rate until at least 1 day after ROP screening.

In the current study, the volume of stool, respiration rate, blood pressure and number of episodes of apnea did not change significantly after ROP screening (p > 0.05 paired-t tests). In a previous report, a significant elevation in systolic blood pressure was observed in 8 of 10 infants [17]. In a different study, percutaneous oxygen saturation and pulse rate changed significantly from baseline values following ROP screening with 2.5% phenylephrine and 0.5% tropicamide eyedrops. There were no significant changes in blood pressure, temperature, or respiration rate [18]. Jiang et al. reported significant increases in blood pressure after administration of phenylephrine 0.5% and tropicamide 0.5% [7]. Lees et al. reported that blood pressure was significantly elevated but pulse rate was unchanged after ROP screening with 2.5% phenylephrine and 0.5% tropicamide eyedrops [13]. Isenberg et al. also reported significantly elevated blood pressures after ROP screening with 2.5% phenylephrine and 0.5% tropicamide eyedrops [12], but blood pressure did not change after ROP screening with the same types and doses of eyedrops in a study by Bolt et al. [19]. Thus, there are still controversies about changes in pulse rate, respiration rate, blood pressure and periods of apnea, which may be due to differences in the kind and/or dose of mydriatics used and/or the time of ophthalmic examination.

The current study had some limitations in that it was a retrospective study, with a small sample size and without control cases. Because we evaluated data only up to 1 day after screening, any long-term effects are unknown. Finally, the cause of the abdominal adverse effects was unclear and not only the mydriatics, but also the ophthalmic examinations themselves, might contribute to the changes seen.

In conclusion, screening for ROP with 0.5% phenylephrine and 0.5% tropicamide eyedrops may have adverse effects on systemic conditions such as abdominal distention, milk consumption and pulse rate. Careful observation for abdominal conditions should be performed after ROP screening with mydriatic.

## Supporting information

S1 File Data set for analysis. (XLSX)
